# GnRH I and II up-regulate MMP-26 expression through the JNK pathway in human cytotrophoblasts

**DOI:** 10.1186/1477-7827-8-5

**Published:** 2010-01-15

**Authors:** Jing Liu, Bin Cao, Yu-xia Li, Xiao-qiu Wu, Yan-ling Wang

**Affiliations:** 1 State Key Laboratory of Reproductive Biology, Institute of Zoology, Chinese Academy of Sciences, Beijing 100101, PR China; 2 College of Environmental and Resource Sciences, Zhejiang University, Hangzhou, 10029, PR China

## Abstract

**Background:**

Matrix metalloproteinase-26 (MMP-26), one of the main mediators of extracellular matrix (ECM) degradation, has been shown to exist in trophoblasts of human placenta and to play a role in trophoblast cell invasion. However, little is known about the regulation of MMP-26 expression in human trophoblasts. Recently, gonadotropin-releasing hormone I (GnRH I) and GnRH II have been shown to regulate the expression of MMP-2, MMP-9/tissue inhibitor of metalloproteinases 1 (TIMP-1), and urokinase plasminogen activator (uPA)/plasminogen activator inhibitor (PAI) in human trophoblasts, suggesting that these two hormones may work as paracrine and/or autocrine regulators in modulating the activities of various protease systems at the feto-maternal interface. In this study, we determined the regulatory effects of GnRH I and GnRH II on the expression of MMP-26 in human immortalized cytotrophoblast-like cell line, B6Tert-1.

**Methods:**

Real-time PCR was used to quantify mRNA levels of MMP-26 in human trophoblast-like cell line, B6Tert-1 and primary cultured cytotrophoblasts. Western blotting was used to characterize the expression of MMP-26 and the phosphorylation of c-Jun NH2-terminal kinase (JNK) and extracellular signal-regulated kinase 1/2 (ERK1/2) in B6Tert-1 cells after treatment with GnRH I and GnRH II.

**Results:**

We found that GnRH I increased MMP-26 expression in B6Tert-1 cells after 12 h of treatment at both the mRNA and protein level, while GnRH II increased MMP-26 expression beginning at 3 h of treatment. Treatment of GnRH I at 1 nM resulted in maximal increase of MMP-26 mRNA and protein levels, whereas GnRH II treatment at a concentration of 100 nM was required to induce maximal increase in MMP-26 expression. In addition, we demonstrated that the activation of JNK, but not ERK1/2, was required for GnRH I and II-stimulated MMP-26 production in B6Tert-1 cells and primary cytotrophoblasts.

**Conclusions:**

These novel findings indicated that GnRH I and II could up-regulate MMP-26 expression through the JNK signaling pathway in human trophoblast-like/trophoblast cells.

## Background

Trophoblast cell invasion is essential for successful implantation and placentation. During the first trimester of gestation, trophoblast cells proliferate, invade the maternal decidua, and differentiate to form chorionic villi, composed of an inner layer of cytotrophoblasts (CTBs) and an outer layer of syncytiotrophoblasts [1,2]. A subpopulation of the proliferating CTBs subsequently streams out of the syncytiotrophoblasts to form mononuclear invasive extravillous cytotrophoblasts (EVTs). These EVTs invade the maternal tissues and penetrate the uterine arterioles, thereby ensuring a continuous blood supply to placenta and the developing fetus [1,2]. These processes require the action of metalloproteinases (MMPs), a family of zinc-dependent proteolytic enzymes that are the main mediators of extracellular matrix (ECM) degradation [3].

MMP-26, also known as endometase or matrilysin-2, was recently identified as the smallest member of MMP family [4-6]. It is widely expressed in villous CTBs, syncytiotrophoblasts and EVTs of human placenta [7]. MMP-26 exhibits wide substrate specificity in cleaving ECM and basement membrane proteins including type IV collagen, fibronectin, fibrinogen, vitronectin and gelatin [6,8]. The fact that most of these ECM components are expressed in human villous trophoblasts [9] suggests the involvement of MMP-26 in the degradation and remodelling of ECM at the feto-maternal interface. In addition, MMP-26 is able to process the lacent MMP-9, to generate its active form [6,10]. Our previous studies revealed that the spatiotemporal expression of MMP-26 was similar to that of MMP-9 in trophoblasts during the first trimester [7,11], implicating that MMP-26 may also indirectly contribute to ECM degradation through activation of proMMP-9 at the feto-maternal interface. Recently, we found that overexpression of MMP-26 increases invasive capacity of human trophoblast cells *in vitro *[12], suggesting the important role of MMP-26 in trophoblast invasion. However, little is known about the regulatory mechanism of MMP-26 expression in human trophoblasts.

An increasing body of evidence has shown the extrapituitary functions of gonadotropin-releasing hormone I (GnRH I) and the second mammalian form of this hormone (GnRH II). In human placenta, GnRH I is widely expressed among distinct subpopulations of trophoblasts throughout gestation [13,14], while GnRH II expression is restricted to mononuclear villous CTBs and EVTs during the first trimester [13]. GnRH receptor (GnRHR) is highly expressed in both CTBs and syncytiotrophoblasts during early gestation [15]. Recently, GnRH I and GnRH II have also been shown to regulate the expression of MMP-2, MMP-9/tissue inhibitor of metalloproteinases 1 (TIMP-1), and urokinase plasminogen activator (uPA)/plasminogen activator inhibitor (PAI) in human EVT cultures [16,17]. These observations suggest the paracrine and/or autocrine regulatory roles of these two hormones in modulating the activities of various protease systems at the feto-maternal interface.

Based on previous findings from our laboratory and others, we hypothesized that GnRH I and GnRH II induce MMP-26 expression in trophoblast cells. In the present study, we investigated the regulatory mechanism of MMP-26 expression by GnRH I and GnRH II using an *in vitro *experimental model of an immortalized human cytotrophoblast-like cell line, B6Tert-1, which has been established in our laboratory [18].

## Methods

### Materials

GnRH I and GnRH II native peptides were obtained from Peninsula Laboratories, Inc. (San Carlos, CA). ERK1/2 inhibitor (PD98059) and JNK inhibitor (SP600125) were purchased from Sigma-Aldrich Corp. (St. Louis, MO). PD98059 and SP600125 were dissolved in DMSO. The antibodies specific against β-actin, phospho-ERK1/2, phospho-JNK, total-ERK1/2 and total-JNK were purchased from Cell Signaling Technology, Inc. (Beverly, MA). The antibodies specific against GnRH receptor were obtained from Lab Vision Corp. (Fremont, CA). The polyclonal antibodies against MMP-26 [5] are kind gifts from Dr. Qing-Xiang A. Sang of Florida State University, Florida, USA.

### Cell culture and treatment

Immortalized human cytotrophoblast-like B6Tert-1 cells were cultured as described previously [18]. In brief, the cells were cultured in collagen I (Cellmatrix Type I-A; Institute of Biochemistry, Osaka, Japan)-coated flasks with a defined serum-free medium [DMEM/F12 medium (Gibco-Invitrogen, San Diego, CA) supplemented with 10 ng/ml epidermal growth factor (EGF; Collaborative Research, Lexington, MA) and 10 mg/ml insulin (Sigma)]. Cells were incubated at 37°C in an atmosphere of 5% CO_2_. To synchronize the cells, at least 18 hours before each hormone treatment, concentration of EGF in the culture medium was reduced from 10 ng/ml to 1 ng/ml and the concentration of insulin was decreased from 10 mg/ml to 1 mg/ml. When reagents were dissolved in DMSO, the same concentration of DMSO was added to medium for the control cells. After pretreatment with inhibitors or vehicle [0.1% (vol/vol) DMSO] for 30 minutes, the cells were treated with GnRH I or GnRH II at concentration of 100 nM for a further 24 hours. Cells were collected at various time points following treatment with GnRH I or GnRH II.

Cells from the human choriocarcinoma cell line, JEG-3, were purchased from American Type Culture Collection (ATCC, Rockville, MD). After thawing, the cells were maintained in DMEM medium (Gibco-Invitrogen) supplemented with antibiotics and 10% (vol/vol) heated-inactivated fetal bovine serum (FBS; Gibco-Invitrogen).

### Isolation of human primary cytotrophoblast (CTB) cells

Human chorionic villi tissues were obtained from patients who underwent therapeutic termination of pregnancy at 6~7 weeks of gestation. Informed consent was provided by the patients, and the project received prior approval from the local ethics committee. The time of gestation was defined according to the first day of the last menstrual period, and further morphological examination by means of stereomicroscope. Primary CTB cells were isolated from chorionic villi tissues as previously described [19]. Briefly, tissues were minced separately and digested with 0.25% (vol/vol) trypsin (Sigma) and DNase I (Sigma). Next, the dispersed cells were washed by DMEM/F12 medium (Gibco-Invitrogen) and then filtered through a nylon sieve to remove the gross villous core residues. The filtered cell suspension was then slowly added to the top of a BSA gradient [prepared by sequential addition of 3%, 2%, and 1% (wt/vol) BSA in DMEM/F12 medium]. The cells were sedimented for 1 h at unit gravity, and cytotrophoblast cells were collected from the bottom of the tube. The purified CTB cells were cultured in defined serum free medium and treated with GnRH I and GnRH II in the absence or presence of inhibitors for 24 h.

### RNA preparation and synthesis of first-strand cDNA

Total RNA was extracted from the B6Tert-1 or CTB cells with TRIzol reagent (Invitrogen, San Diego, CA), according to the manufacturer's instructions. RNA was subjected to DNase I digestion to avoid possible genomic DNA contamination and then reverse transcribed with oligo-dT primers and Superscript II reverse transcriptase (Invitrogen).

### Real-time PCR

The first-strand cDNA generated from the B6Tert-1 or CTB cells served as a template for Real-time PCR using the ABI PRISM 7000 sequence detection system (PerkinElmer Applied Biosystems, Foster City, CA) equipped with a 96-well optical reaction plate. The primers used for SYBR Green Real-time PCR were designed using the PRIMER3 software and were as follows: human MMP-26, 5'-TCC AGC AAG TGC AGA ATG GA-3'(forward) and 5'-GGG CCC ACT GCC AGA AA-3' (reverse); human glyceraldehyde-3-phosphate dehydrogenase (GAPDH), 5'-ATG GAA ATC CCA TCA CCA TCT T-3' (forward) and 5'-CGC CCC ACT TGA TTT TGG-3' (reverse). The primer specificity for MMP-26 and GAPDH was confirmed by running the PCR products on a 2.0% agarose gel. Each Real-time PCR reaction contained 12.5 μl SYBR Green ER qPCR SuperMix (Invirogen), 2.5 μl of primer mixture (400 nm), and 10 μl of cDNA template [10% (vol/vol) RT reaction product] under the following optimized conditions: 50°C for 2 min followed by 95°C for 10 min and 40 cycles of 95°C for 15 sec and 55°C for 1 min. All PCR reactions were performed in triplicate, with the mean being used to determine mRNA levels. Relative mRNA expression levels for MMP-26 were determined using the 2^-ΔΔCT ^method [20] and normalized to the endogenous reference gene, GAPDH.

### Western Blotting analysis

Total protein was isolated from B6Tert-1, JEG-3 or CTB cells using lysis buffer (Cell Signaling). The protein extracted from αT3-1 cells was kindly provided by Dr. Peter C.K. Leung in University of British Columbia, Canada. Total protein (30 μg) was run on 10% SDS-polyacrylamide gels. After electrotransferring the protein to the nitrocellulose membrane (Amersham Pharmacia Biotech, Piscataway, NJ), the membrane was immunoblotted using specific primary antibodies for MMP-26, GnRHR, phospho-ERK1/2 or phospho-JNK at 4°C overnight. The signals were detected with horseradish peroxidase-conjugated secondary antibody (Santa Cruz Biotechnology, Santa Cruz, CA) for 1 hour and visualized using the enhanced chemiluminescence system (Amersham Pharmacia Biotech). After stripping, the membranes were reprobed with β-actin, total-ERK1/2 and total-JNK antibodies, respectively. The relative density of MMP-26 was determined by normalization to the density value of β-actin. The relative densities of phosphorylated forms of ERK1/2 and JNK were normalized to total values of ERK1/2 and JNK. All densities were analyzed using the Gel-Pro Analyzer (software version 4.0; United Bio., Marlton, NJ).

### Transwell insert invasion assay

*In vitro *cell invasion was assayed by determining the ability of cells to invade a synthetic basement membrane. Briefly, 24-well fitted transwell inserts with membranes (8- μm pore size, Millipore Corp, Bedford, MA) were coated with growth factor reduced Matrigel (BD Biosciences, Franklin, NJ) at a concentration of 200 μg/mL and placed in a 24-well plate. B6Tert-1 cells at a concentration of 2 × 10^4 ^were seeded in each insert containing defined medium supplemented with 1 ng/ml EGF and 1 mg/ml insulin and lower chambers were loaded with defined medium containing 10 ng/ml EGF and 10 mg/ml insulin. After incubating with or without GnRH I or GnRH II (100 nM) for 24 h, the cells were then fixed and stained with crystal violet. Non-invaded cells on the upper surface of the membrane were removed using a cotton swab. The membranes were cut from inserts and mounted onto glass slides. The number of stained cells was counted, in at least 15 randomly selected non-overlapping fields of the membranes, using a light microscope.

### Statistical analysis

Data were shown as the mean ± SEM of three individual experiments performed in duplicate or triplicate. Statistical analysis was carried out using one-way ANOVA followed by Dunnett's test, and differences were considered significant for *P *< 0.05. Representative images of Western blot are shown.

## Results

### The characteristics of B6Tert-1 cells

**Figure 1:**
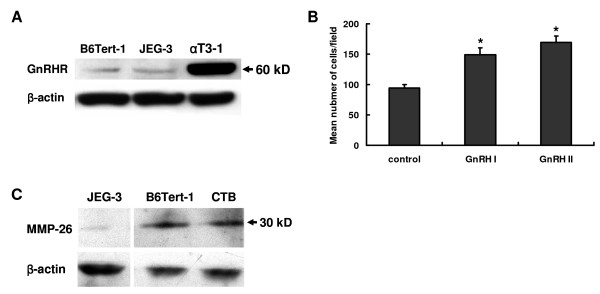
**The characteristics of B6Tert-1 cells**. **A**, Western blotting analyses show the expression of GnRH receptor (GnRHR) in JEG-3 and B6Tert-1 cells. Mouse pituitary gonadotrope αT3-1 cells were used as the positive control. **B**, The B6Tert-1 cells were cultured in the presence of 100 nM GnRH I or GnRH II for 24 h, and their invasive capacities were analyzed by invasion assay. **C**, The expression of MMP-26 protein in JEG-3, B6Tert-1, primary cultured cytotrophoblast (CTB) cells. The data derived from at least three independent sets of experiments (mean ± SEM; n ≥ 3), and the statistic results are presented in the column graphs (*, *P *< 0.05 *vs*. control).

The expression of GnRHR mRNA has been shown in JEG-3 cells and first trimester CTB cells in primary culture [21]. To determine whether the GnRHR gene is expressed in B6Tert-1 cells, levels of GnRHR protein were assessed by Western blotting. As shown in Figure 1A, the expression of GnRHR protein was present in both B6Tert-1 and JEG-3 cells. Mouse pituitary gonadotrope αT3-1 cells, previously shown to express GnRHR [22], were used as the positive control.

To characterize the invasiveness of B6Tert-1 cells in response to GnRH I and II, cells treated with the native peptides of these two subtypes of hormone were subjected to invasion assay. The results indicated that GnRH I and GnRH II increased the invasive capacity of B6Tert-1 cells, with the invasion index increasing to 1.6- and 1.8-fold of control cultures, respectively (Figure 1B). This observation is consistent with our previous report that GnRH I and GnRH II promote EVT cell invasion [23].

The expression of mRNA for MMP-2, MMP-9, MMP-14, TIMP-1, TIMP-2 and TIMP-3 has been shown in B6Tert-1 cells [18], while MMP-26 expression has yet to be identified in this cell line. Western blotting analyses revealed that the expression level of MMP-26 protein in B6Tert-1 cells was comparable to that in the primary CTB cells at 6-7 gestational weeks, but much higher than that of human choriocarcinoma JEG-3 cells (Figure 1C). These results suggest that the B6Tert-1 cell line is a valuable *in vitro *model to study the regulation of GnRH I and II on MMP-26 expression in trophoblast-like/trophoblast cell.

### Time-dependent effects of GnRH I and GnRH II on MMP-26 expression in B6Tert-1 cells

**Figure 2:**
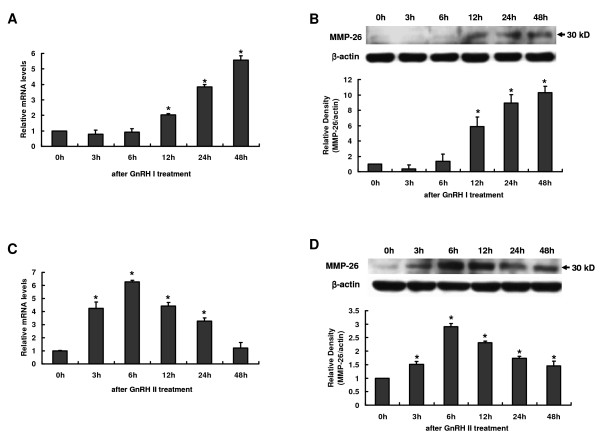
**Time-dependent effects of GnRH I and GnRH II on MMP-26 expression in B6Tert-1 cells**. **A **and **B**, The expression of MMP-26 mRNA (**A**) and protein (**B**) in B6Ter-1 cells cultured with GnRH I (100 nM) for 0, 3, 6, 12, 24 or 48 h. **C **and **D**, The expression of MMP-26 mRNA (**C**) and protein (**D**) in B6Ter-1 cells cultured with GnRH II (100 nM) for 0, 3, 6, 12, 24 or 48 h. Levels of mRNA for MMP-26 in each sample were normalized to the GAPDH at the corresponding time points. Levels of protein for MMP-26 were normalized to the corresponding β-actin. The results derived from both these analyses as well as those from at least three other sets of experiments were standardized to the 0 h control and are represented (mean ± SEM; n ≥ 3) in the bar graphs (*, *P *< 0.05 *vs*. 0 h control).

To investigate the regulatory effects of GnRH I and GnRH II on MMP-26 expression in human cytotrophoblasts, mRNA and protein levels were analyzed from B6Tert-1 cells collected at various time points after treatment with GnRH I or GnRH II (100 nM). Treatment with GnRH I or GnRH II resulted in an increase in MMP-26 production at mRNA or protein level in a time-dependent manner (Figure 2), whereas the addition of vehicle to the culture medium had no effect on the expression of MMP-26 in B6Tert-1 cells at any of the time points examined in this study (data not shown).

GnRH I increased the expression of MMP-26 mRNA in B6Tert-1 cells during the interval of 12 h to 48 h after treatment. The levels of MMP-26 mRNA at 48 h were approximately 5 fold higher than that of the 0 h (Figure 2A). A coordinate increase in the levels of MMP-26 protein in these cell cultures was also observed between 12 h and 48 h after GnRH I (Figure 2B).

GnRH II stimulated MMP-26 expression in B6Tert-1 cells in a manner differential to that of GnRH I: levels for MMP-26 mRNA and protein increased at 3 h after GnRH II treatment, peaked at 6 h, and gradually decreased thereafter (Figure 2C and 2D). By 48 h after treatment, MMP-26 mRNA expression decreased to a level comparable to 0 h, while the protein level remained significantly greater than those detected at 0 h (Figure 2C and 2D).

### Different dosage effects of GnRH I and GnRH II on MMP-26 production in B6Tert-1 cells

**Figure 3:**
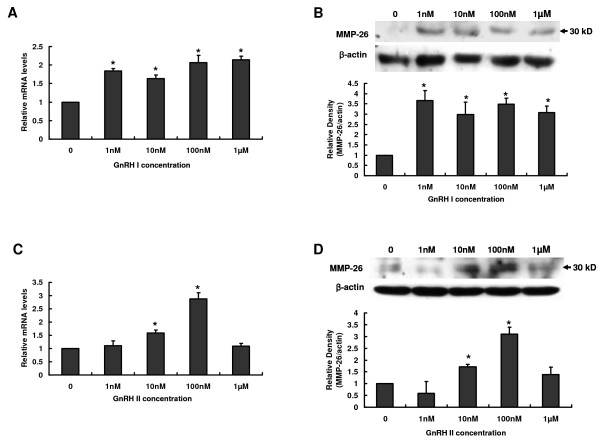
**Different dosage effects of GnRH I and GnRH II on MMP-26 expression in B6Tert-1 cells**. **A **and **B**, The expression of MMP-26 mRNA (**A**) and protein (**B**) in B6Ter-1 cells cultured with increasing concentrations of GnRH I (0, 1, 10, 100 nM or 1 μM) for 24 h. **C **and **D**, The expression of MMP-26 mRNA (**C**) and protein (**D**) in B6Ter-1 cells cultured with increasing concentrations of GnRH II (0, 1, 10, 100 nM or 1 μM) for 24 h. Levels of mRNA for MMP-26 in each sample were normalized to the corresponding GAPDH. Levels of protein for MMP-26 were normalized to the corresponding β-actin. The results derived from both these analyses as well as those from at least three other sets of experiments were standardized to the untreated control and are represented (mean ± SEM; n ≥ 3) in the bar graphs (*, *P *< 0.05 *vs*. untreated control).

To further determine the dosage effects of GnRHs on MMP-26 production, B6Tert-1 cells were treated with GnRH I or GnRH II at concentrations of 1, 10, 100 nM and 1 μM) for 24 h. GnRH I significantly increased MMP-26 mRNA and protein levels in B6Tert-1 cells at all the hormone concentrations examined in these studies. The treatment of 1 nM GnRH I was sufficiently high to induce maximal increase in MMP-26 expression (Figure 3A and 3B). GnRH II increased MMP-26 mRNA and protein levels in B6Tert-1 cells in a dose-dependent manner. A significant increase in MMP-26 expression was first detected at concentration of 10 nM and the highest expression of MMP-26 was observed in B6Tert-1 cells in the presence of 100 nM GnRH II. However, GnRH II at concentration of 1 μM was ineffective (Figure 3C and 3D).

### Involvement of JNK pathway in GnRH I and II-induced MMP-26 production

**Figure 4:**
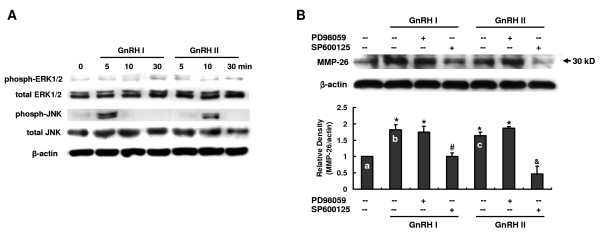
**Involvement of JNK activity in GnRH I and II-induced MMP-26 production**. **A**, B6Tert-1 cells were stimulated with GnRH I or GnRH II (100 nM) for 5, 10 and 30 min, or the cells were left untreated as a control. Phosphorylation of ERK1/2 or JNK and the total amount of ERK1/2 or JNK were determined by Western blotting analyses. **B**. Following 30-minute pretreatment with PD98059 (10 μM, ERK1/2 inhibitor) or SP600125 (10 μM, JNK inhibitor), B6Tert-1 cells were treated with GnRH I or GnRH II (100 nM) for 24 h and the protein levels of MMP-26 analyzed by Western blotting analyses. Levels of protein for MMP-26 were normalized to the corresponding β-actin. The results derived from both these analyses as well as those from at least three other sets of experiments were standardized to the untreated control and are represented (mean ± SEM; n ≥ 3) in the bar graphs (*, *P *< 0.05 *vs*. bar a; #, *P *< 0.05 *vs*. bar b; &, *P *< 0.05 *vs*. bar c).

The mitogen-activated protein kinase (MAPK) signaling cascade has been shown to mediate the action of GnRH in human trophoblast cells [24]. To address the role of the MAPK cascade in regulating MMP-26 expression by GnRH I and GnRH II, we examined the phosphorylation of JNK and ERK1/2 in B6Tert-1 cells treated with GnRH I or GnRH II for 0, 5, 10 and 30 minutes. As shown in Figure 4A, addition of 100 nM of GnRH I or GnRH II resulted in a transient activation of JNK at 5 min or 10 min, respectively (Figure 4A). However, the addition of both GnRH I and GnRH II had no effects on ERK1/2 activity in B6Tert-1 cells (Figure 4A).

To further clarify the involvement of JNK and ERK1/2 in GnRH-induced MMP-26 expression, B6Tert-1 cells were cultured with GnRH I or GnRH II in the absence or presence of the JNK inhibitor (SP600125; 10 μM) or ERK1/2 inhibitor (PD98059; 10 μM). SP600125, but not PD98059, significantly blocked MMP-26 induction (Figure 4B). These data indicate that stimulation of MMP-26 expression by GnRH I and II requires the activation of JNK, but not ERK1/2 signaling molecules.

### Regulatory effects of GnRH I and GnRH II on MMP-26 expression in primary cultured CTBs

**Figure 5:**
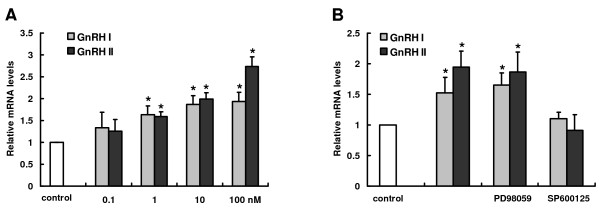
**Regulatory effects of GnRH I and GnRH II on MMP-26 expression in primary cultured CTB cells**. **A**, The expression of MMP-26 mRNA in CTB cells cultured with GnRH I (gray) or GnRH II (black) at concentrations of 0.1, 1, 10 and 100 nM for 24 h. **B**, Following 30-minute pretreatment with PD98059 (10 μM, ERK1/2 inhibitor) or SP600125 (10 μM, JNK inhibitor), CTB cells were incubated with GnRH I (gray) or GnRH II (black) at concentrations of 100 nM for 24 h and the mRNA levels of MMP-26 analyzed by Real-time PCR. Levels of mRNA for MMP-26 in each sample were normalized to the corresponding GAPDH. The results derived from both these analyses as well as those from at least three other sets of experiments were standardized to the untreated control and are represented (mean ± SEM; n ≥ 3) in the bar graphs (*, *P *< 0.05 *vs*. untreated control).

We further confirmed the regulatory effects of GnRH I and GnRH II on MMP-26 expression using the primary isolates of first trimester CTB cells. The cells were incubated with GnRH I and GnRH II at concentrations of 0.1-100 nM for 24 h. A significant increase in MMP-26 mRNA levels was only observed in trophoblast cultured in the presence of higher concentrations of these two hormones (1-100 nM) (Figure 5A). GnRH II at a concentration of 100 nM induced the maximal increase in MMP-26 expression (Figure 5A).

To further determine the signaling mediators involved in GnRH-induced MMP-26 expression, CTB cells were cultured with 100 nM GnRH I and II in the presence of 10 μM SP600125 or PD98059. As shown in Figure 5B, SP600125 inhibited the stimulatory effects of GnRH I and GnRH II on MMP-26 mRNA levels, whereas PD98059 had no effects on MMP-26 expression.

## Discussion

MMP-26 is a recently discovered and only partially characterized human proteinase [4-6]. Unlike all other MMPs, the *Mmp-26 *gene does not exist in the murine genome [4-6]. Besides its extensive distribution in cancer cells of epithelial origin [8], MMP-26 is also restricted in human placenta and uterus, but nor in other normal tissues [4,6]. Previous studies showed its intensive expression in human trophoblasts *in vivo *and invasive-promoting effect on trophoblast cells *in vitro *[7,12], suggesting the unique role of MMP-26 in trophoblast invasion at the feto-maternal interface. However, the regulation of MMP-26 expression in trophoblast cells remains to be determined. In this study, we utilized human trophoblast-like cell line, B6Tert-1, to show that 1) both GnRH I and GnRH II could up-regulate MMP-26 expression, 2) GnRH I and GnRH II had differential effects on MMP-26 expression, and 3) GnRH I and II-induced MMP-26 expression was mediated by JNK, but not ERK1/2 signaling pathway.

The B6Tert-1 cell line is an immortalized cytotrophoblast-like cell line developed from normal human placental villi during the first trimester. These cells are transfected with human telomerase catalytic subunit gene (*htert*) and thus maintain reconstituted telomerase activity [18]. B6Tert-1 cells exhibit the characteristics of normal EVTs by producing various biomarkers including CK8, HLA-G, integrin α1 and integrin β1 [18]. They also exhibit the ability to invade matrix and express proteinase genes including MMP-2, MMP-9, MMP-14, TIMP-1, TIMP-2 and TIMP-3, which are important properties of EVTs [18]. In this study, we further demonstrated that the expression of MMP-26 in B6Tert-1 cells was comparable to that in the primary CTB cells at early gestation. The invasiveness of B6Tert-1 cells was regulated by paracrine and/or autocrine factors such as EGF and transforming growth factor β [18]. Here we showed that the invasive ability of B6Tert-1 cells was increased by GnRH I and GnRH II. This observation is also consistent with our previous report that GnRH I and GnRH II stimulated the invasiveness of primary EVT cells [23]. Our data and other groups' studies strongly suggest that the B6Tert-1 cell line is a valuable *in vitro *cellular model for the investigation of trophoblast-like/trophoblast cell behaviors [25,26]. In the present study, we utilized B6Tert-1 cells to determine the regulatory mechanism of GnRHs on MMP-26 expression in human trophoblast-like/trophoblast cells.

The classical mammalian GnRH (GnRH I) is a decapeptide that is well-known for its role in regulating the release of gonadotropins from the pituitary [27]. Evidence shows that in addition to its classical endocrine functions, GnRH I has direct regulatory actions on the development and function of gonads and other reproductive tissues such as the ovary, endometrium and placenta [16,17,27-29]. A distinct gene encoding a second form of GnRH, termed GnRH II, that is expressed in human extrapituitary tissues [30], has been shown to imitate the paracrine/autocrine function of GnRH I in extrapituitary compartments [16,17,27-29]. Recent studies demonstrated that GnRH I and GnRH II promoted the invasive capacity of human trophoblasts by regulating MMP-2, MMP-9/TIMP-1 and uPA/PAI protease systems [16,17,23]. Consistent with these findings, our present work indicated that both GnRH I and GnRH II were capable of increasing the mRNA and protein levels of MMP-26 in trophoblast-like/trophoblast cells. In addition to its direct proteolytic action on ECM substrates, MMP-26 has also been reported to be an activator of proMMP-9 in prostate cancer cells and correlated to MMP-9 activity in esophageal carcinoma [10,31]. Considering the similar expression patterns between MMP-26 and MMP-9 in trophoblasts during early gestation [7,11], GnRH-induced MMP-26 production may, at least in part, participate in proteolysis of the ECM through activating the latent form of MMP-9. The coordinate induction of MMP-2, MMP-9 and MMP-26 by GnRHs suggests that these enzymes possibly elicit their functions as components of a proteolytic cascade in trophoblasts.

Although GnRH II is capable of mimicking the biological actions of GnRH I in extrapituitary tissues, evidence shows differential effects of these two hormones. For instance, GnRH II exhibited more potent anti-proliferative effects than an equimolar dose of GnRH I in human endometrial and ovarian cancer cells [32]. In human placenta, GnRH I was more effective than GnRH II on hCG synthesis and secretion [33], while GnRH II appeared to be more potent than GnRH I in stimulating leptin secretion [34]. Recently, Chou et al. demonstrated that GnRH II was capable of eliciting its regulatory effects on MMP/TIMP systems at lower hormone concentrations than GnRH I in human EVTs [17]. In this study, our data showed that the time course of the stimulatory effect of GnRH II on MMP-26 expression was earlier than that of GnRH I. In addition, GnRH I and GnRH II had different dosage effects on MMP-26 expression in B6tert-1 cells. These differential actions of GnRH I and GnRH II in regulating MMP-26 expression might be explained by different degradation pathways [35], different signaling cascades [23], different receptor affinities [36] or even different types of GnRH receptor [17,23].

It has been well established in gonadotropic cells that the binding of GnRH with its receptor activates MAPK cascades, leading to phosphorylation of ERK1/2 and JNK [37]. This process is an important link for the transmission of GnRH signals from the cell surface to the nucleus [37]. MAPK also has been shown to mediate the action of GnRH in human trophoblast cells [24]. In the present study, we demonstrated the rapid and transient activation of JNK by GnRH I and GnRH II in B6Tert-1 cells. GnRH I and II-induced MMP-26 expression was blocked by specific inhibition of JNK, but not ERK1/2 in B6Tert-1 and primary CTB cells. JNK signaling has been shown to be involved in the production of various MMPs that promote invasiveness and migration of cancer cells [38-40]. A recent study showed that JNK, but neither ERK1/2 nor p38 MAPK, was critical for GnRH-mediated production of MMP-2 and MMP-9 in human ovarian cancer cells [41]. In agreement with these observations, our data suggests that JNK, but not ERK1/2, may play a central role in mediating the effects of GnRHs on MMP-26 production in human trophoblast-like/trophoblast cells.

## Conclusions

In summary, this is the first report in our knowledge to demonstrate that GnRH I and GnRH II up-regulate the expression of MMP-26 through the JNK pathway in human trophoblast-like/trophoblast cells. These findings provide the basis for further studies into the regulatory mechanism of MMP-26 expression and its biological function in human trophoblasts during implantation and placentation.

## Competing interests

The authors declare that they have no competing interests.

## Authors' contributions

JL and YLW designed the study. JL and BC performed the research. YXL isolated human primary cytotrophoblast cells. XQW performed the Western blotting analyses of Figure 1C. JL and YLW interpreted the results and drafted the manuscript. All authors read and approved the final manuscript.
